# Evaluation of *TRIM63* RNA in situ hybridization (RNA-ISH) as a potential biomarker for alveolar soft-part sarcoma (ASPS)

**DOI:** 10.1007/s12032-024-02305-9

**Published:** 2024-02-23

**Authors:** Alexander S. Taylor, Rahul Mannan, Liron Pantanowitz, Arul M. Chinnaiyan, Saravana M. Dhanasekaran, Steven Hrycaj, Xuhong Cao, May P. Chan, David Lucas, Xiao-Ming Wang, Rohit Mehra

**Affiliations:** 1 Department of Pathology, University of Michigan Medical School, 2800 Plymouth Road, Building 35, Ann Arbor, MI 48109 USA; 2 Michigan Center for Translational Pathology, Ann Arbor, MI USA; 3 Rogel Cancer Center, Michigan Medicine, Ann Arbor, MI USA; 4 Department of Urology, University of Michigan Medical School, Ann Arbor, MI USA; 5 Howard Hughes Medical Institute, Ann Arbor, MI USA

**Keywords:** Alveolar soft-part sarcoma, Pediatric sarcoma, Renal cell carcinoma, RNA in situ hybridization, Pecoma, Melanoma, Diagnostic biomarker, *TRIM63*

## Abstract

Alveolar soft-part sarcoma (ASPS) is a rare soft tissue tumor with a broad morphologic differential diagnosis. While histology and immunohistochemistry can be suggestive, diagnosis often requires exclusion of other entities followed by confirmatory molecular analysis for its characteristic *ASPSCR1-TFE3* fusion. Current stain-based biomarkers (such as immunohistochemistry for cathepsin K and TFE3) show relatively high sensitivity but may lack specificity, often showing staining in multiple other entities under diagnostic consideration. Given the discovery of RNA in situ hybridization (RNA-ISH) for *TRIM63* as a sensitive and specific marker of MiTF-family aberration renal cell carcinomas, we sought to evaluate its utility in the workup of ASPS. *TRIM63* RNA-ISH demonstrated high levels (H-score greater than 200) of expression in 19/20 (95%) cases of ASPS (average H-score 330) and was weak or negative in cases of paraganglioma, clear cell sarcoma, rhabdomyosarcoma, malignant epithelioid hemangioendothelioma, as well as hepatocellular and adrenal cortical carcinomas. Staining was also identified in tumors with known subsets characterized by *TFE3* alterations such as perivascular epithelioid cell neoplasm (PEComa, average H-score 228), while tumors known to exhibit overexpression of TFE3 protein without cytogenetic alterations, such as melanoma and granular cell tumor, generally showed less *TRIM63* ISH staining (average H-scores 147 and 96, respectively). Quantitative assessment of *TRIM63* staining by RNA-ISH is potentially a helpful biomarker for tumors with molecular TFE3 alterations such as ASPS.

**Supplementary Information:**

The online version contains supplementary material available at 10.1007/s12032-024-02305-9.

## Introduction

Alveolar soft-part sarcoma (ASPS) is a rare malignant translocation-associated soft tissue neoplasm that can arise in the deep soft tissue of the extremities, trunk (retroperitoneum, pelvis), as well as the head and neck, and less frequently in the female genital tract, mediastinum, bone, urinary bladder, and other viscera [1]. ASPS can occur at any age, with a range of 1–78 years. However, young individuals are more susceptible to developing ASPS, with a median age of 25 years. In fact, 72% of patients who develop ASPS are younger than 30 years old. Female gender and head and neck tumors are more common in infants and children. [2]. ASPS usually presents as a slowly growing painless mass. Distant metastasis (often arising late, 10 or more years after primary diagnosis/resection) is not uncommon. Often the metastases of ASPS are slow-growing and minimally destructive, allowing some patients to experience long-term survival even with metastatic disease [2–5].

**Fig. 1:**
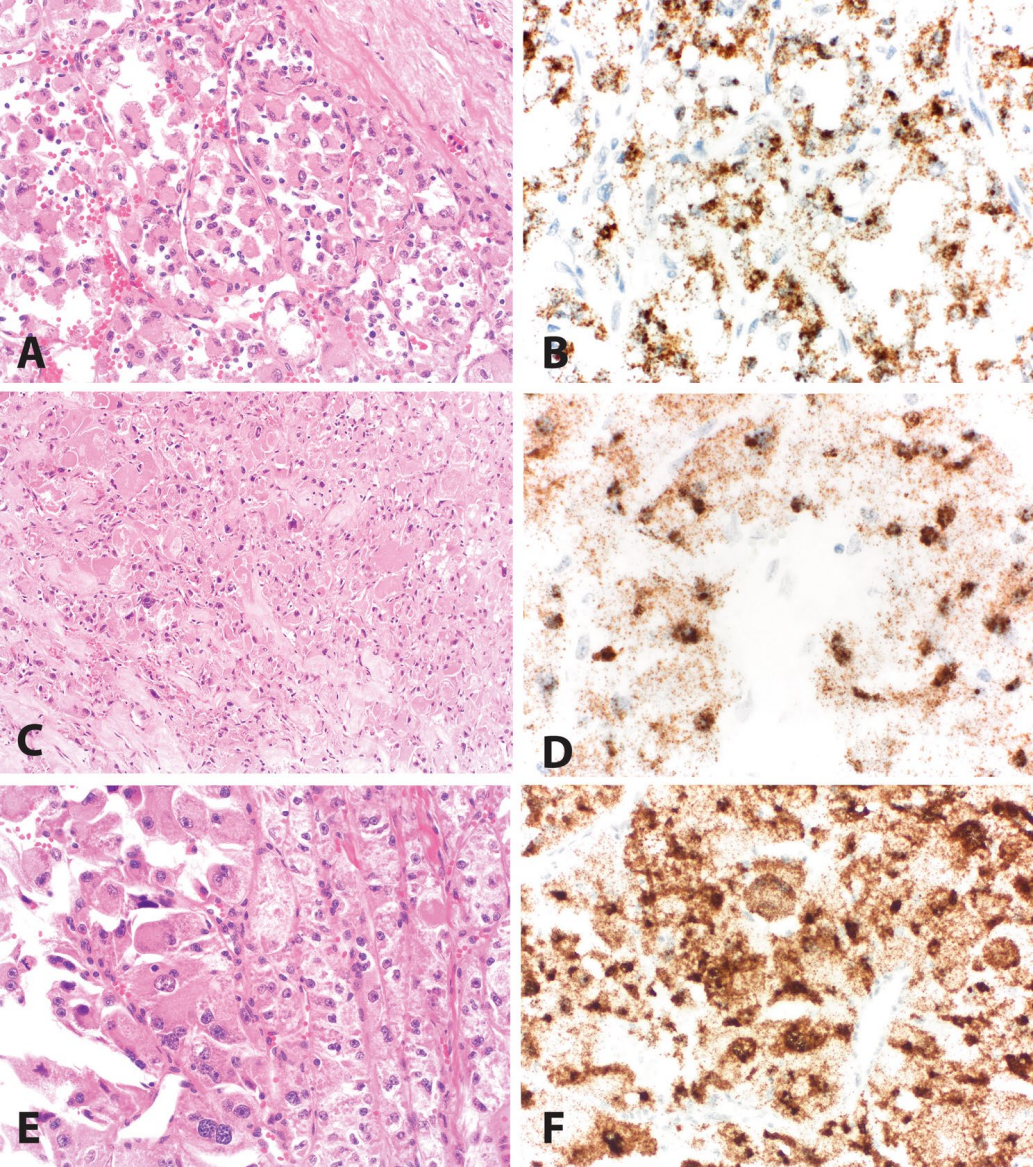
Alveolar soft-part sarcoma (ASPS). **A** ASPS with alveolar nests lined by vaguely discohesive epithelioid cells with abundant eosinophilic cytoplasm. **B** Positive TRIM63 RNA-ISH staining of case in panel A (H-score 295). **C** ASPS with abundant granular eosinophilic cytoplasm and foci of infiltrative, spindled growth. **D** Positive TRIM63 RNA-ISH staining of case in panel C (H-score 313) **E** ASPS with heterogenous features including foci (left) with marked pleomorphism and rhabdoid/hepatoid cytomorphology. F. Positive TRIM63 RNA-ISH staining of case in panel E (H-score 395). *A, C, and E: Hematoxylin and eosin. Magnifications 100X (A), 200X (A, E), and 400X (B, D, F)*

**Fig. 2:**
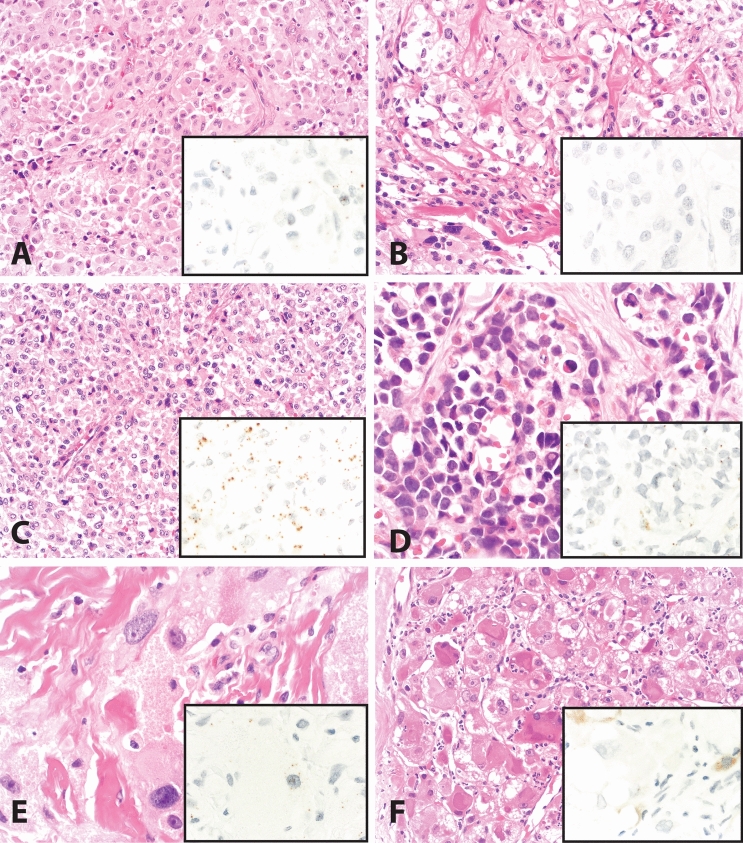
Differential considerations in the diagnostic workup of ASPS. **A** Melanoma, with nests of discohesive epithelioid cells; TRIM63 RNA-ISH (inset) shows scattered rare staining (H-score = 20). **B** Paraganglioma, with nests of epithelioid cells separated by delicate branching vasculature; TRIM63 RNA-ISH (inset) shows scattered rare staining (H-score = 20). **C** Epithelioid PEComa with nested and focal alveolar architecture and cells with prominent eosinophilic to clear cytoplasm. TRIM63 RNA-ISH (inset) shows dot-like expression with more cytoplasmic staining than nuclear staining (H-score 195). D. Alveolar rhabdomyosarcoma, with nests and alveolar spaces lined by cells with abundant clear to eosinophilic cytoplasm; TRIM63 RNA-ISH (inset) shows scattered rare staining (H-score = 4). E. Granular cell tumor, with voluminous granular eosinophilic cytoplasm; TRIM63 RNA-ISH (inset) shows scattered rare staining (H-score = 70). F. Hepatocellular carcinoma, with rhabdoid features including abundant eosinophilic cytoplasm; TRIM63 RNA-ISH (inset) shows focal faint cytoplasmic blush-like staining interpreted as nonspecific (H-score = 0). *A–F Hematoxylin and eosin. Magnifications: 200X (A–C, F) and 400X (D, E, and all insets)*

The histogenesis of ASPS remains incompletely understood, but the entity is now molecularly defined by a recurrent unbalanced translocation der(17)t(X;17)(p11;q25) resulting in *ASPSCR1-TFE3* fusion, activating the *MET* proto-oncogene signaling pathway [6–8]. ASPS is characterized histologically by large, polygonal tumor cells that contain abundant eosinophilic cytoplasm and PASD-positive crystals, arranged in a nested, alveolar, or pseudovascular growth pattern (Fig. 1). Reaching a histologic diagnosis of ASPS can be challenging, given its rarity and morphologic overlap with other epithelioid, myogenic, and hepatoid diagnostic considerations. These include, but are not limited to, various types of tumors such as perivascular epithelioid cell neoplasm (PEComa), paraganglioma, rhabdomyoma, rhabdomyosarcoma, granular cell tumor, clear cell sarcoma, hepatocellular carcinoma, adrenal cortical adenoma/carcinoma, and melanoma. (Fig. 2) [1, 4].

The data regarding the immunohistochemistry (IHC) for TFE3 protein are variable in the literature for ASPS, with positive staining in 92% of ASPS in one series of 24 cases [9] and 100% in another series of 18 cases [10]. In the two published series above, tumors that lacked TFE3 fusions were used as controls. However, some level of positive staining was detected in various cases of high-grade myxofibrosarcoma, malignant peripheral nerve sheath tumor, granular cell tumor, chordoma, adrenal cortical carcinoma, and distal bile duct carcinoma. [9, 10]. In a research study focused on ASPS, 22 cases were analyzed, and found that 91% of them had TFE3 staining. However, it was also observed that some of the control cases of paraganglioma, adrenal cortical carcinoma, and granular cell tumor also showed positive staining. These control cases are often considered diagnostically similar to ASPS due to their similar appearance. [11]. A heads-on comparison of staining between ASPS and granular cell tumors showed TFE3 expression in 91% of granular cell tumors [12]. Anecdotally, our experience with TFE3 IHC in the setting of renal tumor classification has followed similar patterns, with difficulty interpreting moderate expression as specific or nonspecific. As concluded by Tsuji et al*.* [9], molecular methods for detecting the pathognomonic gene fusion transcript of ASPS have superior sensitivity and specificity compared with TFE3 IHC; hence, we decided to explore RNA-ISH technology which can identify m-RNA transcripts under light/bright microscopy and can be performed in a clinical surgical pathology lab. Similarly, cathepsin K IHC has demonstrated impressive sensitivity for ASPS, staining 100% of 18 cases of ASPS in a series focused on tumors with *TFE3* gene fusions [13]. However, while carcinomas (without TFE3 fusions) are usually cathepsin K negative, mesenchymal neoplasms can frequently show cathepsin K staining (e.g., more than half of 414 non-ASPS mesenchymal tumors in one series), including many cases of melanoma (which is also within the morphologic differential diagnosis for ASPS) [14].

Transcriptomic data of ASPS samples have identified *TRIM63* (*tripartite motif containing* 63) as a possible biomarker for ASPS [15]. Given its regulation by *TFE3* and *TFEB* [16], *TRIM63* RNA in situ hybridization (RNA-ISH) is currently clinically performed in our department, with primary use by genitourinary pathologists to detect MiTF-family aberration associated (formerly referred to as “translocation-associated”) renal cell carcinomas. As part of prior work to discover and establish this biomarker as a sensitive and specific test in the context of renal tumor classification [17], we evaluated a limited number of cases of ASPS, which were found to demonstrate strong and diffuse RNA-ISH *TRIM63* expression. We were intrigued by this observation as currently, no known biomarkers by immunohistochemistry or RNA in situ hybridization (RNA-ISH) can reliably detect ASPS. The importance of *TRIM63* overexpression as a potential biomarker for ASPS fueled us to perform these interrogations.

In this study, we sought to quantitatively evaluate *TRIM63* RNA-ISH in a cohort of ASPS cases, and select other tumors with similar morphology, to assess the performance of *TRIM63* RNA-ISH for ASPS and to highlight diagnostic scenarios in which *TRIM63* RNA-ISH may have utility and clinical value for distinguishing and/or confirming a diagnosis of ASPS. In addition, we provide further rationale for the current study by illustrating the enrichment of *TRIM63* in ASPS and TRIM63 as a downstream target of TFE3 by in-silico analysis of publicly available data.

## Materials and methods

### Case selection

With approval by the institutional review board (IRB), the laboratory information system of an academic medical center was queried to identify cases of ASPS, in addition to other diagnostic entities that may be in the differential diagnosis, some of which potentially harbor shared molecular aberrations (*TFE3* fusion) or transcriptomic patterns (TFE3 overexpression without gene fusion). Cases with available tissue blocks and slides were collated, and diagnoses were confirmed by histologic review. Tested cases include ASPS (*n* = 21), paraganglioma (*n* = 3), PEComa (*n* = 5), granular cell tumor (*n* = 3), melanoma (*n* = 11), rhabdomyosarcoma (*n* = 8), rhabdomyoma (*n* = 1), adrenal cortical tumors (adenomas and carcinomas, *n* = 3), hepatocellular carcinoma (*n* = 3), clear cell sarcoma (*n* = 2), and malignant epithelioid hemangioendothelioma (*n* = 3).

### RNA in situ hybridization (RNA-ISH)

RNA-ISH was performed on whole formalin-fixed, paraffin-embedded (FFPE) 4-micron-thick tissue sections using the RNAscope VS Universal HRP kit (Advanced Cell Diagnostics, Newark, CA) and target probe against *TRIM63* (532299 Hs-TRIM63 targeting NM_032588.3 270-1396nt) on Discovery Ultra-automated slide-staining system (Roche-Ventana Medical Systems). Signals were developed using the mRNA DAB detection kit (Roche-Ventana Medical Systems). RNA quality was evaluated using a positive control probe targeting the human housekeeping gene *PPIB* as described previously [18]. The assay background was monitored using a negative control probe targeting the bacillus subtilis *DapB* gene. All the critical reagents and probe details have been presented in Supplementary Table 1.

### H-score calculation

RNA-ISH staining slides were examined under 100× and 200×magnification for RNA signals in tumor cells. The RNA-ISH signals were quantified according to previously validated RNAscope scoring methodology: score 0 = no staining or < 1 dot per 10 cells; score 1 = 1–3 dots per cell; score 2 = 4–9 dots per cell and no or very few dot clusters; score 3 = 10–15 dots per cell and < 10% dots in clusters; score 4 =  > 15 dots per cell and > 10% dots in clusters. H-score was calculated for each examined tissue section as the sum of the percentage of cells with RNAscope score 0–4: (A% × 0) + (B% × 1) + (C% × 2) + (D% × 3) + (E% × 4), A + B + C + D + E = 100.

### Cross-validatory in-silico meta-analysis of ASPS data sets

For direct biological validation, we utilized a publicly available ASPS gene expression meta-analysis dataset utilized by Stockwin et al. [19]. This meta-analysis comprised 4 publicly available ASPS gene expression datasets (from GEO database https://www.ncbi.nlm.nih.gov/geo/), which includes GSE68591 (contains microarray dataset of ASPS cell line ASPS-1) [19], GSE13433 (microarray dataset from 7 ASPS patients) [15], GSE32569 (microarray dataset from 3 ASPS patients) [20], GSE5 4729 (RNAseq dataset from 5 ASPS patients) datasets [21]. We identified up-regulated genes (fold change > 2) in ASPS samples versus control samples shared among all five datasets and used them as ASPS signature. We extracted fold-change ASPS signature genes including *TRIM63* and visualized them in bar plot and heatmap.

In addition, to identify the TRIM63 as a downstream of TFE3, we employed the publicly available gene expression data from a recent publication by Tanaka et al. [22], where they knocked down the TFE3 gene by siRNA in human ASPS1 cell line and generated gene expression microarray data (GSE215316). GEO2R (https://www.ncbi.nlm.nih.gov/geo/geo2r/) was used to perform differential analysis between siTFE3 and control samples. We then tested the enrichment of the ASPS signature defined upon TFE3 knockdown with fgsea R package [23].

### Statistical analysis

Unpaired two-tailed *t* test was used to compare the mean H-scores between ASPS and other tumor types with positive staining.

## Results

### High TRIM63 RNA-ISH expression in ASPS

**Fig. 3:**
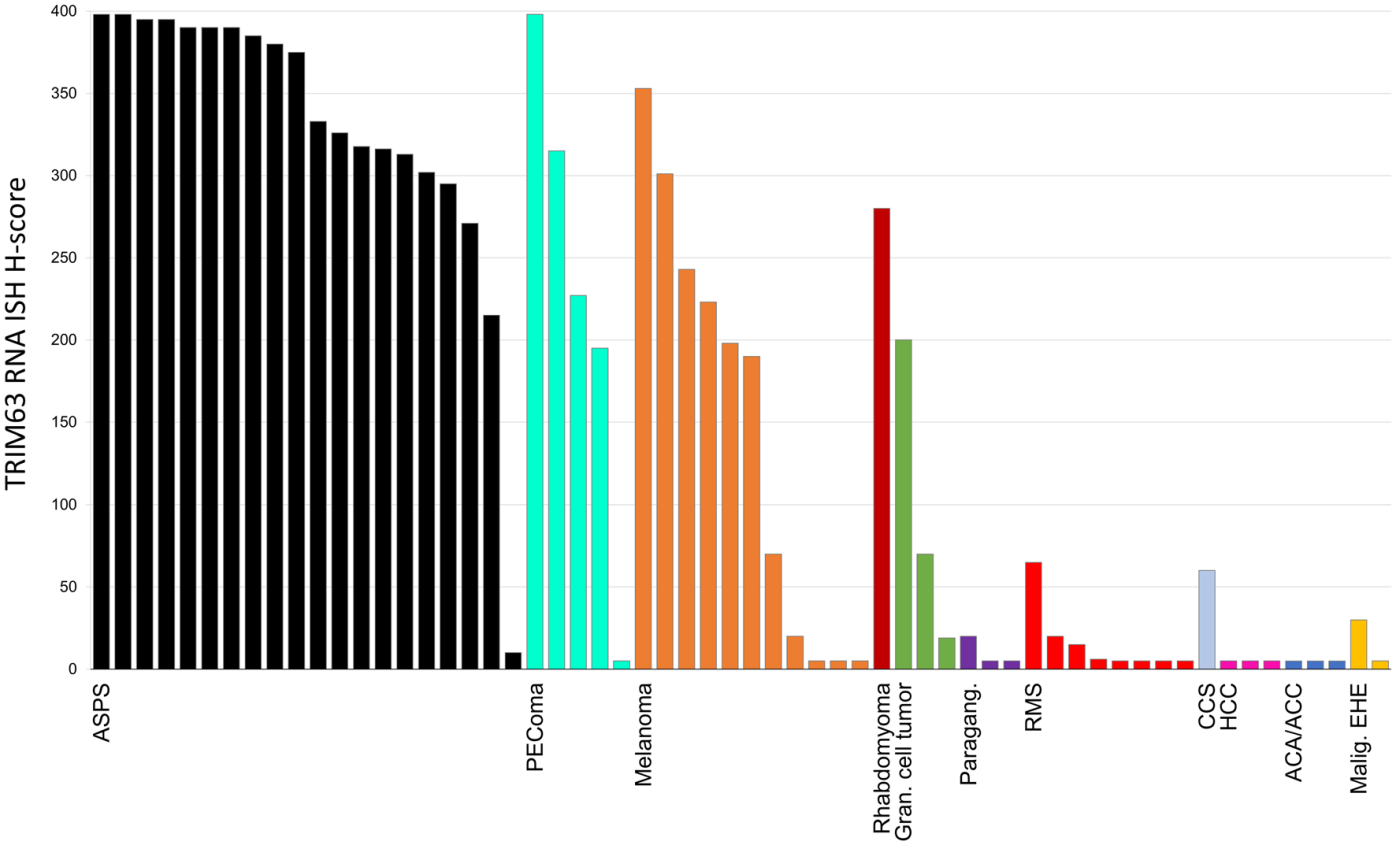
*TRIM63* RNA-ISH staining in ASPS and other tested tumors. H-scores are calculated as described in the methods section. H-scores of less than five (including zero) were plotted as “5” so that each case could be visually identified on the chart. The colors of the bars are only for visual distinction between tested entities. *ASPS* alveolar soft-part sarcoma; *PEComa* perivascular epithelioid cell neoplasm, *Gran*. granular, *Paragang*. paraganglioma, *CCS* clear cell sarcoma, *RMS* rhabdomyosarcoma, *HCC*:hepatocellular carcinoma, *ACA/ACC* adrenal cortical adenoma/adrenal cortical carcinoma, *Malig. EHE* malignant epithelioid hemangioendothelioma

*TRIM63* semiquantitative scores (H-scores) for all cases are plotted in Fig. 3. *TRIM63* RNA-ISH expression was seen in all 20 successfully assayed cases of ASPS (average H-score 330), although one case that had undergone decalcification before processing showed only weak staining (H-score = 25). The positive control (*PPIB* probe) failed in one ASPS case due to poor RNA quality, and the case was excluded from further analysis. Of the 20 resulting cases, 19 (95%) showed an H-score of greater than 200, and 16 (80%) showed an H-score of greater than 300. *TRIM63* m-RNA transcripts in the RNA-ISH were seen as strong, punctate brown dots in both the nucleus and cytoplasm of the tumor cells. Examples of staining showing strong positivity for *TRIM63* RNA-ISH in an ASPS section are seen in Fig. 1. Nontumoral tissue generally demonstrated negative staining, except for low-level expression in cases with adjacent benign skeletal muscle.

### Absent to low expression of TRIM63 RNA-ISH in a majority of other (non-ASPS) tumors

Absent (H-score of 0) or weak staining (H-score of 100 or less) was noted in all successfully assayed cases of paraganglioma, malignant epithelioid hemangioendothelioma, rhabdomyosarcoma, clear cell sarcoma, hepatocellular carcinoma, and adrenal cortical adenoma/carcinoma. Positive control staining (*PPIB*) failed in one case of clear cell sarcoma and one case of malignant epithelioid hemangioendothelioma, which were excluded from final analyses.

### Moderate TRIM63 RNA-ISH expression in PEComa, melanoma, and granular cell tumor

Average *TRIM63* RNA-ISH H-scores for PEComa (228), melanoma (146), and granular cell tumor (96) were generally lower than staining observed in ASPS (*p* = 0.06 for PEComa, *p* < 0.0001 for melanoma, and *p* = 0.0005 for granular cell tumor). Three (of 5) cases of PEComa showed H-scores greater than 200. Cases of granular cell tumor and melanoma tended to show more moderate levels of expression; H-scores exceeded 200 in 4 of 11 melanomas and exceeded 300 in only two of those cases. All three granular cell tumors showed staining less than or equal to 200. In addition, one case of rhabdomyoma included in the current study demonstrated significant *TRIM63* expression (H-score 280).

### In-silico meta-analysis shows TRIM63 is enriched in ASPS cell lines

*TRIM63* expression fold changes (ASPS versus respective control samples) across different ASPS datasets represented as bar plots clearly show overexpression of *TRIM63* in ASPS across all datasets. (Supplementary Fig. 1).

### Gene set enrichment analysis (GSEA) shows TRIM63 is dysregulated in TFE knockdown ASPS cell line

**Fig. 4:**
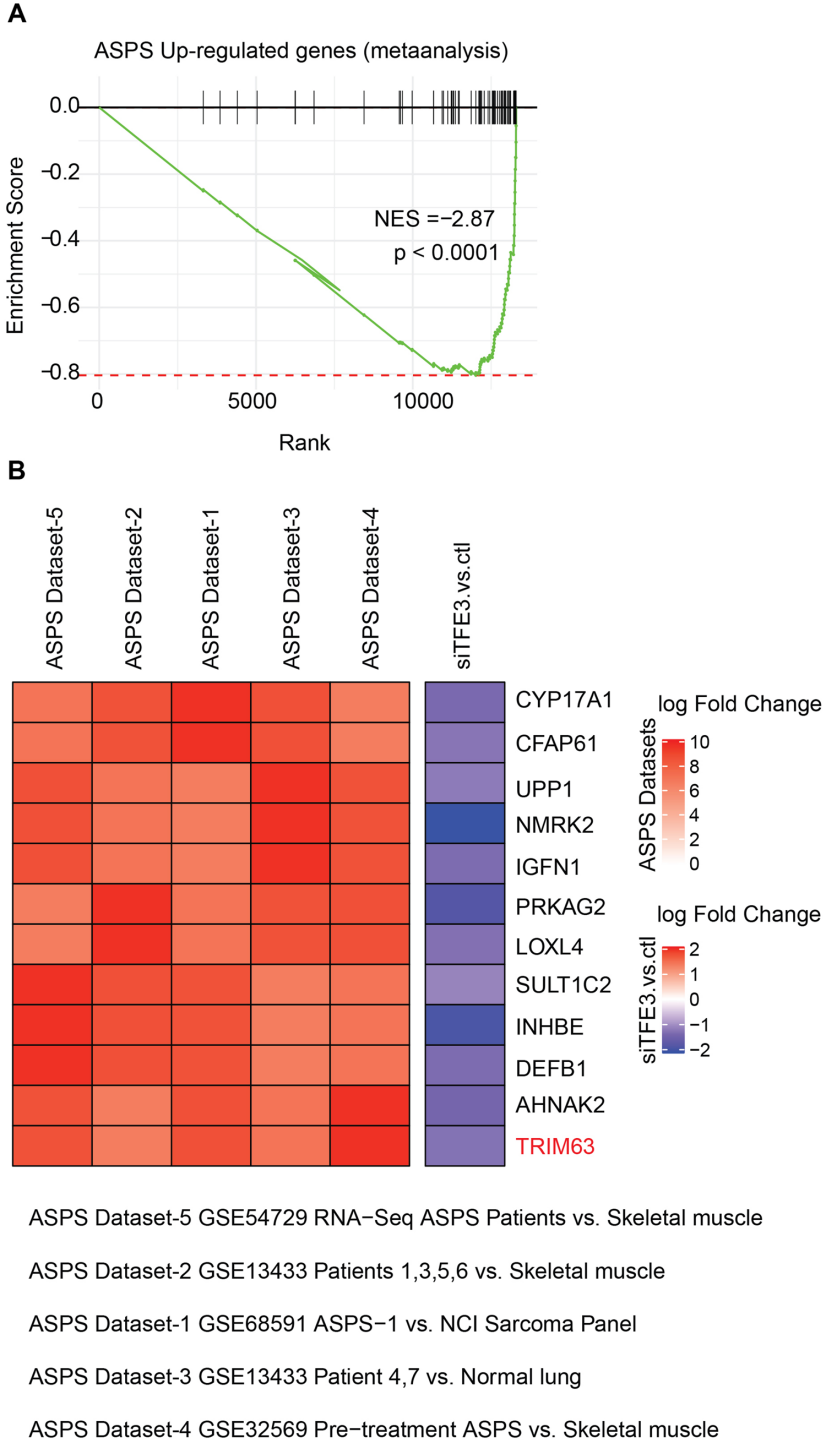
TRIM63 is transcriptionally regulated by TFE3. **A** Gene set enrichment analysis of ASPS tumor-upregulated genes show significant negative enrichment in TFE3 knockdown gene expression data. **B** Heatmap represents the log2-fold changes of gene expression in ASPS tumors compared to indicated control samples in various publicly available datasets as reported earlier. Besides TRIM63, several ASPS-upregulated genes are downregulated upon TFE3 knockdown (siTFE3 vs control column) indicating potential transcriptional regulation of these target genes by TFE3

We identified genes upregulated in ASPS tissues supported by multiple published datasets (see Methods) and performed GSEA analysis to show that besides TRIM63, several other ASPS markers are also downstream targets of TFE3 (showing downregulation in TFE3 knockdown with siRNA) (Fig. 4).

## Discussion

ASPS is a rare disease and a challenging diagnosis to render without cytogenetic testing. Nevertheless, accurate diagnosis is important, as disease management is currently evolving. Targeted therapies toward VEGF, MET signaling, and immune modulation are being studied, and while some clinical trials are agnostic of subclassification in treating soft tissue sarcomas, growing familiarity with recurrent molecular features and implicated pathogenic pathways have led to clinical trials being designed specifically for advanced ASPS (e.g., atezolizumab and bevacizumab, in NCT03141684) [24, 25]. Accurate and efficient diagnosis of ASPS is also crucial to accrue patients with this rare tumor type. There is morphologic overlap between ASPS and other tumors with similar presentations, and as discussed above, other promising stain-based biomarkers for ASPS such as TFE3 and cathepsin K have limitations (with unfortunately low specificity when comparing entities commonly considered along with ASPS), leading to the need for a more specific biomarker. Additionally, Ki67, while an important proliferation marker, is highly non-specific in the diagnostic setting for ASPS. In the literature, the results are unclear but there is a suggestion that Ki-67 staining may be a prognostic indicator for the development of metastases in ASPS [26].

*TRIM63* is a gene that produces an E3 ubiquitin ligase (also known as muscle-specific RING finger protein 1 [MuRF1]) found in skeletal, cardiac, and smooth muscle [27]. This protein is involved in the ubiquitination and subsequent proteasomal degradation of muscle proteins as regulated by metabolic pathways and has been implicated in the regulation of muscle atrophy hypertrophic cardiomyopathy [28].

Analysis performed on publicly available data sets revealed that *TRIM63* was over-expressed in all five data sets that we curated for the present study. Additionally, work conducted by Stockwin et al. [19] in their in vitro studies (cell line data), that among all the different cell lines, and then focusing on the myogenesis-related transcripts only *TRIM63* was specifically expressed in the ASPS cell line. Interestingly the same authors have provided q-PCR validation of *TRIM63* overexpression in their dataset which shows the specificity of *TRIM63* expression. Thus, supporting the rationale of initiating this biomarker study having a translational value in soft tissue tumor/sarcoma patient management.

**Fig. 5:**
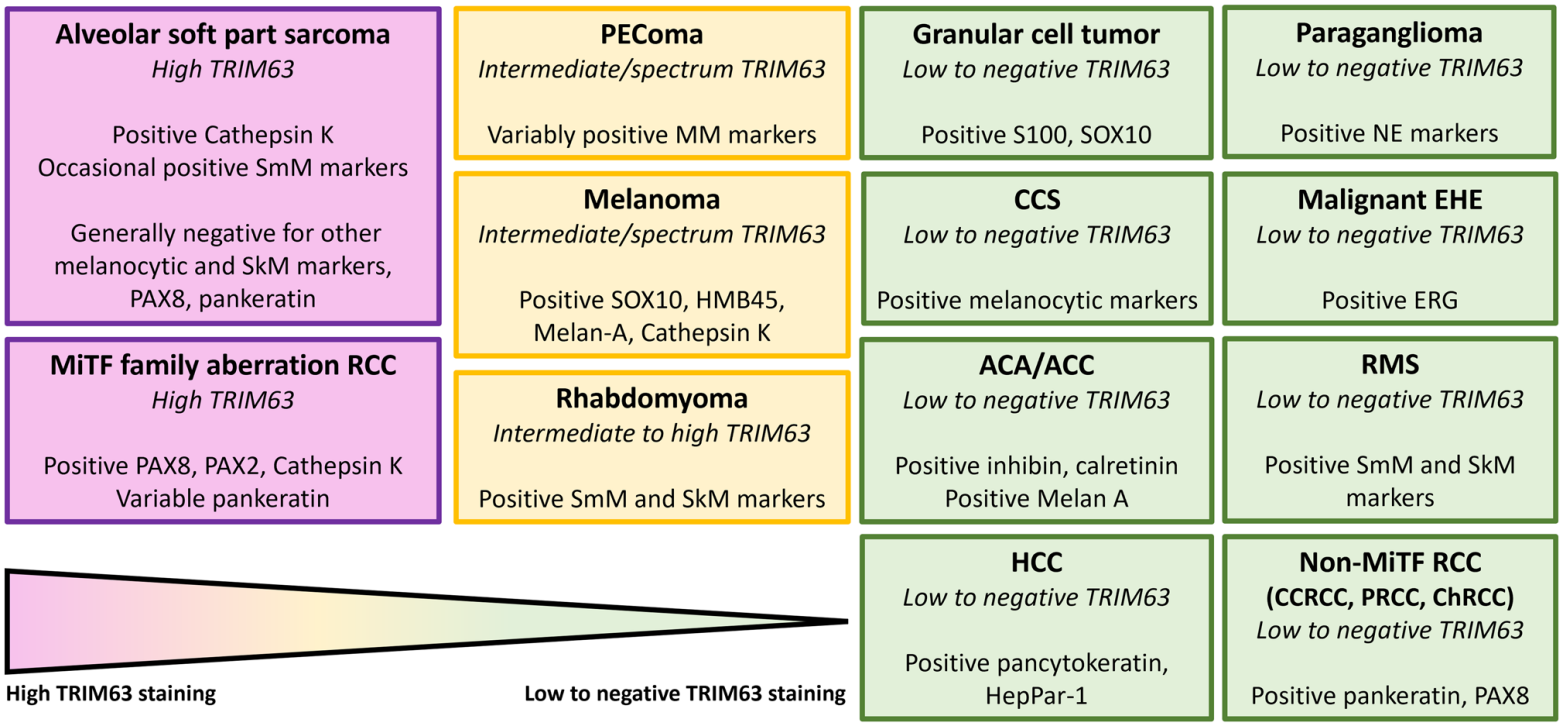
Spectrum of *TRIM63* RNA-ISH expression for use in conjunction with other immunohistochemical studies. Conceptual organization of spectrum of *TRIM63* staining patterns observed in this study and previous work [17] with additional helpful immunostaining patterns. Of particular use are the breadth and strength of myogenic and melanocytic differentiation in differentiating alveolar soft-part sarcoma and MiTF-family aberration RCC (with high *TRIM63* expression, colored in purple) from PEComa, melanoma, and rhabdomyoma (colored in yellow). *RCC* renal cell carcinoma,* PEComa* perivascular epithelioid cell neoplasm,* CCS* clear cell sarcoma, *ACA/ACC* adrenal cortical adenoma/adrenal cortical carcinoma,* HCC* hepatocellular carcinoma, *EHE* malignant epithelioid hemangioendothelioma,* RMS* rhabdomyosarcoma, *CCRCC* clear cell renal cell carcinoma, *PRCC* papillary renal cell carcinoma,* ChRCC* chromophobe renal cell carcinoma

We show *TRIM63* overexpression by RNA-ISH technology to be highly enriched within ASPS, with high levels of expression (H-score greater than 200) in 19/20 (95%) cases. We also identified high levels of *TRIM63* RNA-ISH staining in three of five (60%) PEComas, a subset of which are known to harbor *TFE3* fusions. In supplemental data published with previous work [17], an additional 8 cases of PEComa (renal angiomyolipoma [AML]) underwent *TRIM63* RNA-ISH, with H-scores in epithelioid AML (*n* = 3) averaging 157 (range 66–277) and in classic AML (*n* = 5) averaging 245 (range 149–365). PEComas with *TFE3* fusions are enriched for epithelioid morphology (“epithelioid PEComa”) and alveolar architecture [28, 29], representing substantial morphologic overlap with ASPS. In addition, the emerging molecular overlap between these two tumors makes the unequivocal distinction complicated (both conceptually and practically) as recently described “PEComa-like neoplasms characterized by ASPSCR1-TFE3 fusion” can show morphology that more closely resembles PEComa than ASPS (despite their identical pairs of translocation partners) [30]. In the current study, the average *TRIM63* RNA-ISH H-scores in PEComa were less than that of ASPS (228 and 330, respectively), but not statistically significantly so; subsets of PEComa and ASPS may exist on a spectrum of TFE3-related mesenchymal neoplasia that cannot always be fully delineated using current tools. That said, broad and/or strong immunohistochemical expression of myogenic and melanocytic markers may further help resolve such situations. In Fig. 5 we elaborate on the patterns of IHC that may be helpful when used in conjunction with *TRIM63* RNA-ISH; in particular, a moderate H-score for *TRIM63* RNA-ISH in combination with broad (multiple marker) and strong myomelanocytic marker staining would favor a diagnosis of PEComa over ASPS.

Tortola et al. demonstrated that *TRIM63* expression is tightly regulated by a transcriptional network comprised of the protein kinase D (PKD)-family members and the class IIa histone deacetylases as well as the MiT/TFE family members TFEB and TFE3. Utilizing luciferase assays performed on COS-7 cell line extracts transfected with Hs_TRIM63-Luc with increasing the amount of TFEB and TFE3 the authors were able to conclusively show an over-expression of TRIM63 in a statistically significant manner [16]. Therefore, *TRIM63* RNA-ISH staining would be expected in not only TFE3 fusion-associated neoplasms (like ASPS) but also in some non-TFE3-fused neoplasms. [12, 31–34]. Thus, given the regulation of *TRIM63* by TFE3, positive *TRIM63* RNA-ISH staining can be expected. Similarly, in melanoma, *TRIM63* and *CAPN3* are a direct target of *MiTF*, wherein *TRIM63* mRNA expression was positively correlated with MiTF transcription factor and knockdown of *MiTF* decreased TRIM63 levels [35, 36].

In our cohort, while tumors that have *MiTF* pathway activation without *TFE3* rearrangement (i.e., granular cell tumor and melanoma) demonstrated *TRIM63* RNA hybridization, they generally did so at a lower level than that of ASPS, with lower average H-scores (*p* < 0.05). Based on this data, we propose an H-score threshold value of greater than 200 to consider ASPS, and more specific threshold value of greater than 300 that is more suggestive of *TFE3*-rearranged neoplasia such as ASPS (or *TFE3*-rearranged PEComa, or translocation-associated RCC) over other considerations such as melanoma, granular cell tumor, and other tested entities. These quantitative thresholds in a calibrated assay would offer some objectivity and additional specificity that is lacking when interpreting TFE3 [9–11] and cathepsin K [13, 14] IHC in the workup of cases of ASPS for which tumors like granular cell tumor and melanoma remain in the differential consideration. Further research (with more control cases, additional immunohistochemical evaluation, and, importantly, correlative/confirmatory molecular testing) will be necessary to establish the validity of these proposed thresholds. As conceptualized in Fig. 5, there is utility of quantitative *TRIM63* RNA-ISH in combination with a practical panel of immunohistochemical stains (which would further help rule in or rule out melanoma or granular cell tumor); for example, robust expression of multiple melanocytic markers in the presence of only intermediate *TRIM63* RNA-ISH expression would favor a diagnosis of melanoma.

*TRIM63* RNA-ISH staining in a single case diagnosed as rhabdomyoma may reflect overexpression of constitutive gene transcripts, as *TRIM63* is expressed in normal skeletal muscle [27, 28]; indeed, in cases with nontumoral/adjacent benign skeletal muscle, low-level TRIM63 RNA-ISH staining was observed. Multi-institutional collaboration may be required to accumulate enough cases for further testing and conclusions, as rhabdomyomas are an important clinical and histologic consideration, especially in the workup of pediatric oro-maxillofacial tumors.

This study has several strengths. First, it used a novel molecular technology (RNA-ISH) and a biomarker on a focused cohort comprising molecular and morphological mimics. Second, whole tissue sections enabled a thorough assessment of heterogenous biomarker expression in both tumor and adjacent tissue. However, one limitation of TRIM63 as a diagnostic marker is its dependence on a complex scoring system and threshold to differentiate between ASPS and non-ASPS tumors. Additional work including multi-institutional collaboration may be needed to strengthen the aforementioned proposed thresholds.

In summary, *TRIM63* RNA-ISH may be useful in challenging cases, not necessarily as a replacement for broad sarcoma-specific molecular panels, but as a cost-effective adjunct stain in cases for which ASPS is one of several differential considerations, allowing for more efficient selection of cases for ancillary molecular testing. Familiarization with this assay for its potential inclusion in a panel of stains for working up epithelioid soft tissue neoplasms may allow for a more streamlined diagnostic process for patients with these rare tumors.

### Supplementary Information

Below is the link to the electronic supplementary material.

Supplementary file1 (JPG 819 KB) Overexpression of TRIM63 across 5 different data sets

Supplementary file2 (DOCX 13 KB) Details of reagents and critical commercial assays

## Data Availability

The datasets generated during and/or analyzed during the current study are available from the corresponding author on reasonable request.

## References

[1] Jaber OI, Kirby PA (2015). Alveolar soft part sarcoma. Arch Pathol Lab Med.

[2] Jambhekar NA, Ladanyi M. Alveolar Soft Part Sarcoma. In: Fletcher CMD, ed. Soft tissue and bone tumors. Lyon (France): International Agency for Research on Cancer 2020

[3] Wang HT, Jacobson A, Harmon DC, Choy E, Hornicek FJ, Raskin KA, Chebib IA, DeLaney TF, Chen YLE (2016). Prognostic factors in alveolar soft part sarcoma: a SEER analysis. J Surg Oncol.

[4] Ordonez NG (1999). Alveolar soft part sarcoma: a review and update. Adv Anat Pathol.

[5] Portera CA, Ho V, Patel SR, Hunt KK, Feig BW, Respondek PM, Yasko AW, Benjamin RS, Pollock RE, Pisters PW (2001). Alveolar soft part sarcoma: clinical course and patterns of metastasis in 70 patients treated at a single institution. Cancer.

[6] Heimann P, Devalck C, Debusscher C, Sariban E, Vamos E (1998). Alveolar soft-part sarcoma: further evidence by FISH for the involvement of chromosome band 17q25. Genes Chromosomes Cancer.

[7] Joyama S, Ueda T, Shimizu K, Kudawara I, Mano M, Funai H, Takemura K, Yoshikawa H (1999). Chromosome rearrangement at 17q25 and xp11.2 in alveolar soft-part sarcoma: a case report and review of the literature. Cancer.

[8] Ladanyi M, Lui MY, Antonescu CR, Krause-Boehm A, Meindl A, Argani P, Healey JH, Ueda T, Yoshikawa H, Meloni-Ehrig A, Sorensen PH, Mertens F, Mandahl N, van den Berghe H, Sciot R, Dal Cin P, Bridge J (2001). The der(17)t(X;17)(p11;q25) of human alveolar soft part sarcoma fuses the TFE3 transcription factor gene to ASPL, a novel gene at 17q25. Oncogene.

[9] Tsuji K, Ishikawa Y, Imamura T (2012). Technique for differentiating alveolar soft part sarcoma from other tumors in paraffin-embedded tissue: comparison of immunohistochemistry for TFE3 and CD147 and of reverse transcription polymerase chain reaction for ASPSCR1-TFE3 fusion transcript. Hum Pathol.

[10] Argani P, Lal P, Hutchinson B, Lui MY, Reuter VE, Ladanyi M (2003). Aberrant nuclear immunoreactivity for TFE3 in neoplasms with TFE3 gene fusions - A sensitive and specific immunohistochemical assay. Am J Surg Pathol.

[11] Rekhi B, Ingle A, Agarwal M, Puri A, Laskar S, Jambhekar NA (2012). Alveolar soft part sarcoma 'revisited': clinicopathological review of 47 cases from a tertiary cancer referral centre, including immunohistochemical expression of TFE3 in 22 cases and 21 other tumours. Pathology.

[12] Chamberlain BK, McClain CM, Gonzalez RS, Coffin CM, Cates JM (2014). Alveolar soft part sarcoma and granular cell tumor: an immunohistochemical comparison study. Hum Pathol.

[13] Martignoni G, Gobbo S, Camparo P, Brunelli M, Munari E, Segala D, Pea M, Bonetti F, Illei PB, Netto GJ, Ladanyi M, Chilosi M, Argani P (2011). Differential expression of cathepsin K in neoplasms harboring TFE3 gene fusions. Modern Pathol.

[14] Zheng G, Martignoni G, Antonescu C, Montgomery E, Eberhart C, Netto G, Taube J, Westra W, Epstein JI, Lotan T, Maitra A, Gabrielson E, Torbenson M, Iacobuzio-Donahue C, Demarzo A, Shih IM, Illei P, Wu T, Argani P (2013). A broad survey of cathepsin K immunoreactivity in human neoplasms. Am J Clin Pathol.

[15] Stockwin LH, Vistica DT, Kenney S, Schrump DS, Butcher DO, Raffeld M, Shoemaker RH (2009). Gene expression profiling of alveolar soft-part sarcoma (ASPS). BMC Cancer.

[16] Pablo Tortola C, Fielitz B, Li Y, Rudebusch J, Luft FC, Fielitz J (2020). Activation of tripartite motif containing 63 expression by transcription factor EB and transcription factor binding to immunoglobulin heavy chain enhancer 3 is regulated by protein kinase D and class IIa histone deacetylases. Front Physiol.

[17] Wang XM, Zhang YP, Mannan R, Skala SL, Rangaswamy R, Chinnaiyan A, Su FY, Cao XH, Zelenka-Wang S, McMurry L, Xiao H, Spratt DE, Sangoi AR, Shao LN, Betz BL, Brown N, Tickoo SK, McKenney JK, Argani P, Gupta S, Reuter VE, Chinnaiyan AM, Dhanasekaran SM, Mehra R (2021). TRIM63 is a sensitive and specific biomarker for MiT family aberration-associated renal cell carcinoma. Modern Pathol.

[18] Mannan R, Wang X, Bawa PS, Zhang Y, Skala SL, Chinnaiyan AK, Dagar A, Wang L, Zelenka-Wang SB, McMurry LM, Daniel N (2023). Characterization of intercalated cell markers KIT and LINC01187 in chromophobe renal cell carcinoma and other renal neoplasms. Int J Surg Pathol.

[19] Stockwin LH (2020). Alveolar soft-part sarcoma (ASPS) resembles a mesenchymal stromal progenitor: evidence from meta-analysis of transcriptomic data. PeerJ.

[20] Kummar S, Allen D, Monks A, Polley EC, Hose CD, Ivy SP, Turkbey IB, Lawrence S, Kinders RJ, Choyke P, Simon R (2013). Cediranib for metastatic alveolar soft part sarcoma. J Clin Oncol.

[21] Goodwin ML, Jin H, Straessler K, Smith-Fry K, Zhu JF, Monument MJ, Grossmann A, Randall RL, Capecchi MR, Jones KB (2014). Modeling alveolar soft part sarcomagenesis in the mouse: a role for lactate in the tumor microenvironment. Cancer Cell.

[22] Tanaka M, Chuaychob S, Homme M, Yamazaki Y, Lyu R, Yamashita K, Ae K, Matsumoto S, Kumegawa K, Maruyama R, Qu W (2023). ASPSCR1: TFE3 orchestrates the angiogenic program of alveolar soft part sarcoma. Nat Commun.

[23] Sergushichev A (2016). An algorithm for fast preranked gene set enrichment analysis using cumulative statistic calculation. BioRxiv.

[24] Fujiwara T, Nakata E, Kunisada T, Ozaki T, Kawai A (2022). Alveolar soft part sarcoma: progress toward improvement in survival? A population-based study. BMC Cancer.

[25] O'Sullivan Coyne G, Naqash AR, Sankaran H, Chen AP (2021). Advances in the management of alveolar soft part sarcoma. Curr Probl Cancer.

[26] Sanjuan X, Sobel ME, Yang J, Merino MJ (2000). Alveolar soft part sarcoma: the role of prognostic markers. Ann Diagn Pathol.

[27] The GTEx Consortium (2023) TRIM63. The Genotype-Tissue Expression (GTEx) Project. GTEx Portal: https://gtexportal.org/home/gene/TRIM63: The Broad Institute of MIT and Harvard

[28] Bodine SC, Baehr LM (2014). Skeletal muscle atrophy and the E3 ubiquitin ligases MuRF1 and MAFbx/atrogin-1. Am J Physiol Endocrinol Metab.

[29] Argani P, Aulmann S, Illei PB, Netto GJ, Ro J, Cho HY, Dogan S, Ladanyi M, Martignoni G, Goldblum JR, Weiss SW (2010). A distinctive subset of PEComas harbors TFE3 gene fusions. Am J Surg Pathol.

[30] Schoolmeester JK, Dao LN, Sukov WR, Wang L, Park KJ, Murali R, Hameed MR, Soslow RA (2015). TFE3 translocation-associated perivascular epithelioid cell neoplasm (PEComa) of the gynecologic tract: morphology, immunophenotype, differential diagnosis. Am J Surg Pathol.

[31] Argani P, Wobker SE, Gross JM, Matoso A, Fletcher CDM, Antonescu CR (2022). PEComa-like neoplasms characterized by ASPSCR1-TFE3 fusion: another Face of TFE3-related mesenchymal neoplasia. Am J Surg Pathol.

[32] Chamberlain BK, McClain CM, Gonzalez RS, Coffin CM, Cates JM (2015). Granular cell tumors overexpress TFE3 without corollary gene rearrangement–Reply. Hum Pathol.

[33] Liu Y, Zheng Q, Wang C, Wang J, Ming J, Zhang Y, Li X, Cho WC, Wang L, Li QC, Qiu XS, Wang EH (2019). Granular cell tumors overexpress TFE3 without gene rearrangement: Evaluation of immunohistochemistry and break-apart FISH in 45 cases. Oncol Lett.

[34] Schoolmeester JK, Lastra RR (2015). Granular cell tumors overexpress TFE3 without corollary gene rearrangement. Hum Pathol.

[35] Dilshat R, Fock V, Kenny C, Gerritsen I, Lasseur RMJ, Travnickova J, Eichhoff OM, Cerny P, Moller K, Sigurbjornsdottir S, Kirty K, Einarsdottir BO, Cheng PF, Levesque M, Cornell RA, Patton EE, Larue L, de Tayrac M, Magnusdottir E, Ogmundsdottir MH, Steingrimsson E (2021). MITF reprograms the extracellular matrix and focal adhesion in melanoma. Elife.

[36] Rambow F, Job B, Petit V, Gesbert F, Delmas V, Seberg H, Meurice G, Van Otterloo E, Dessen P, Robert C, Gautheret D, Cornell RA, Sarasin A, Larue L (2015). New functional signatures for understanding melanoma biology from tumor cell lineage-specific analysis. Cell Rep.

