# Supplementary figures and images for: Evaluation of TRIM63 RNA in situ hybridization (RNA-ISH) as a potential biomarker for alveolar soft-part sarcoma (ASPS)
Source: Med Oncol. 2024 Feb 23;41(3):76. doi: 10.1007/s12032-024-02305-9 (PMC10891236; doi:10.1007/s12032-024-02305-9)

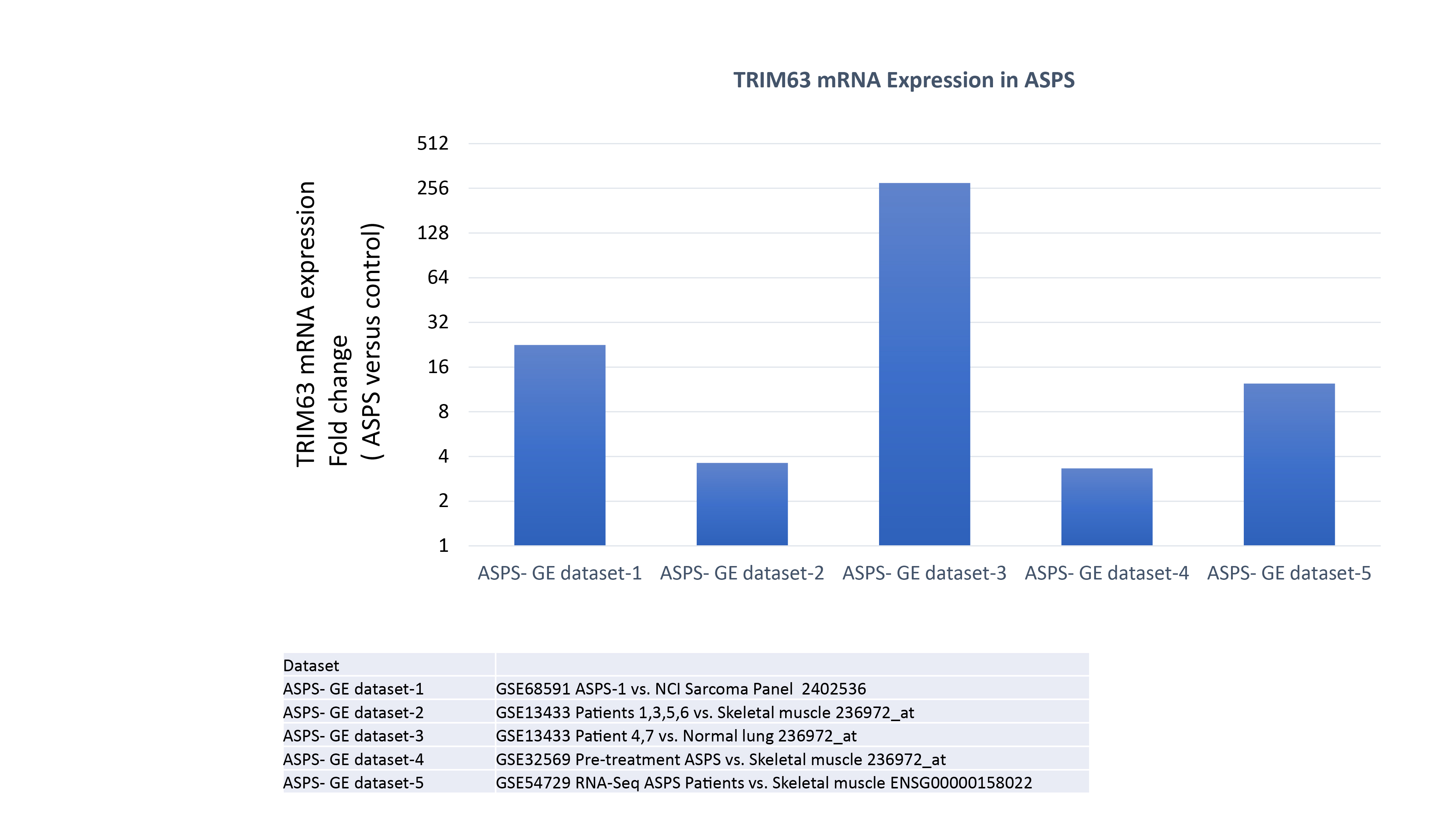

Supplement: Supplementary file 1 — Supplementary file1 (JPG 819 KB) Overexpression of TRIM63 across 5 different data sets [file 12032_2024_2305_MOESM1_ESM.jpg]
