# Supplementary material for: Evaluation of TRIM63 RNA in situ hybridization (RNA-ISH) as a potential biomarker for alveolar soft-part sarcoma (ASPS)
Source: Med Oncol. 2024 Feb 23;41(3):76. doi: 10.1007/s12032-024-02305-9 (PMC10891236; doi:10.1007/s12032-024-02305-9)
Supplement: Supplementary file 2 — Supplementary file2 (DOCX 13 KB) Details of reagents and critical commercial assays [file 12032_2024_2305_MOESM2_ESM.docx]

**Supplementary Table 1 Details of reagents and critical commercial assays**

| **Reagent** | **Source** | **Identifier** |
| --- | --- | --- |
| **RNA-ISH Probes** | | |
| RNAscope Target Probe - Hs-TRIM63 | Advanced Cell Diagnostics, Inc | Catalog No. 532299 |
| RNAscope Positive Probe - Hs-PPIB | Advanced Cell Diagnostics, Inc | Catalog No. 313901/313909 |
| RNAscope Negative Probe – DapB | Advanced Cell Diagnostics, Inc | Catalog No. 310043/312039 |
| **Critical Commercial Assays** | | |
| Discovery CC1 | Roche-Ventana Medical System | Catalog No. 950-500 |
| Discovery CC2 | Roche-Ventana Medical System | Catalog No. 950-123 |
| RNAscope® 2.5 HD Reagent Kit -BROWN | Advanced Cell Diagnostics, Inc | Catalog No. 322300 |
| RNAscope® VS Universal HRP Reagent Kit | Advanced Cell Diagnostics, Inc | Catalog No. 323200 |
| Discovery mRNA DAB Detection RUO | Roche-Ventana Medical System | Catalog No. 760-224 |
